# Virulence and Genomic Profiles of *Klebsiella pneumoniae* Isolated from Pediatric Patients in Henan, China (2021–2023)

**DOI:** 10.1007/s11596-025-00137-w

**Published:** 2025-11-17

**Authors:** Yue Qiu, Chu-ning Wang, Jun-wen Yang, Kai-jie Gao, Jun-zhen Zhu, Bao-liang Wang, He Tian, Yi-bing Cheng, Mei Zeng

**Affiliations:** 1 Department of Infectious Diseases, Children’s Hospital of Fudan University, Shanghai, 201102 China; 2 Department of Clinical Laboratory, Children’s Hospital Affiliated to Zhengzhou University, Henan Children’s Hospital, Zhengzhou Children’s Hospital, Zhengzhou, 450018 China; 3 Department of Emergency, Children’s Hospital Affiliated to Zhengzhou University, Henan Children’s Hospital Zhengzhou Children’s Hospital, Zhengzhou, 450018 China

**Keywords:** Carbapenem-resistant *Klebsiella pneumoniae*, Hypervirulent genes, Children, Virulence, Genomic epidemiology

## Abstract

**Objective:**

This study aimed to investigate the virulence characteristics of *Klebsiella pneumoniae* (KP) strains isolated from children to analyze the genetic relatedness between pediatric and local adult CRKP isolates and to identify clinical risk factors associated with high-risk strains.

**Methods:**

KP strains and corresponding clinical data were collected at a tertiary provincial children's hospital in Henan province from January 2021 to May 2023. The molecular and clinical characteristics of pediatric carbapenem-resistant KP (CRKP) strains were analyzed. Genomic data from local adult isolates were integrated, and the virulence profiles of pediatric and adult isolates were compared. Clinical risk factors for isolating high-risk strains were initially screened via LASSO regression and then evaluated via multivariate binomial regression.

**Results:**

Among the 205 collected KP isolates, 87 (42.4%) were CRKP, and 118 (57.6%) were carbapenem-sensitive KP (CSKP). The predominant carbapenem resistance gene was *bla*_KPC-2_ (89.7%), followed by *bla*_NDM-1_ (5.7%) and *bla*_IMP-4_ (4.6%). Ten sequence types (STs) were identified among the CRKP isolates, with ST11–KL47–KPC2 (52.9%) being the predominant genotype. Screening for virulence genes revealed that 55 (63.2%) CRKP isolates carried both the hypervirulence-associated genes *iuc* and *rmpA2*. Single-nucleotide polymorphism (SNP) analysis revealed that 34 of these strains had fewer than 10 SNPs. Phylogenetic and population genetic analyses revealed close genomic relatedness between pediatric and adult CRKP strains. Young age and exposure to invasive procedures were identified as independent risk factors for the isolation of *iuc*^+^*rmpA2*^+^ CRKP.

**Conclusions:**

The ST11–KL47–KPC2 genotype was the predominant CRKP isolate in pediatric patients. The close genomic relatedness between pediatric and adult CRKP isolates suggests a common ancestor that has disseminated across populations. The high prevalence and clonal transmission of pediatric *iuc*^+^*rmpA2*^+^ CRKP strains warrant heightened clinical vigilance.

**Supplementary Information:**

The online version contains supplementary material available at 10.1007/s11596-025-00137-w.

## Introduction

*Klebsiella pneumoniae* (KP) is a ubiquitous opportunistic pathogen and a major cause of nosocomial infections and community-acquired infections [1]. The increasing prevalence of carbapenem-resistant KP (CRKP) over the past two decades has become a significant clinical issue [2]. CRKP is listed by the World Health Organization (WHO) as a critical priority pathogen because of its ability to transfer resistance genes, cause severe infections, and impose a substantial disease burden [3]. Carbapenemase production is the primary molecular mechanism underlying CRKP emergence, particularly involving KP carbapenemase (KPC) and metallo-β-lactamase (MBL). Understanding these molecular mechanisms facilitates the selection of appropriate, newly developed antimicrobial agents [4]. CRKP infections are more prevalent among neonatal patients than among adults in China [5]. Although KPC-producing KP isolates are predominant in adults, several studies have reported a higher prevalence of NDM-producing KP isolates in pediatric patients [6, 7]. Clonal dissemination of CRKP is associated with several distinct strain types. ST258 is commonly reported worldwide, whereas ST11, considered the most transmissible clone, contributes significantly to the increasing prevalence of CRKP in China, accounting for over 80% of clinical CRKP strains in recent years [8, 9].

In addition to antimicrobial resistance, the emergence and spread of hypervirulent KP (hvKP) have raised significant concerns since 2011. These strains exhibit increased virulence, leading to life-threatening community- and hospital-acquired infections [10]. The pathogenesis of hvKP involves multiple virulence genes, including *iuc*, *iro*, and *ybt* (biosynthesis of aerobactin, salmochelin, and yersiniabactin), *rmpA* and *rmpA2* (regulators of the mucoid phenotype), and *peg-344* (a metabolic determinant) [10, 11]. In recent years, two key phenotypes—hypervirulence and carbapenem resistance—have converged in KP, resulting in the emergence of hv-CRKP [12]. This pathogen was first reported in Beijing in 2015 [13]. Genomic evidence suggests that hv-CRKP evolved from CRKP through the acquisition of pLVPK-like virulence plasmids and hypervirulence-associated genes [14]. Notably, an ST11–KL64 CRKP subclone carrying plasmid-associated hypervirulence genes has undergone rapid regional expansion among adult patients in China since 2016 [15, 16], contributing to the dissemination of hypervirulence-associated genes. In contrast, ST11–KL64 CRKP is uncommon in pediatric populations; instead, ST11–KL47 and ST48-KL62 CRKP have been identified as representative clonal lineages in Shanghai [6]. However, surveillance studies of the molecular mechanisms and virulence profiles of pediatric CRKP strains remain limited. Among pediatric patients, hvKP infections were first reported in Japan in 2016 [17], followed by subsequent cases in Russia [18], Malaysia [19], India [20], and Egypt [21]. Since 2021, hvKP has been reported in pediatric cohorts, with prevalence rates of 8.2% (26/319) in Beijing [22], 8.8% (3/34) in Wuhan [23], and 23.6% (83/352) in Shanghai [24]. Understanding recent epidemiological trends in hypervirulence-associated genes in pediatric KP strains can increase awareness and infection control among pediatric patients. Therefore, this retrospective study aimed to characterize the carbapenemase genotypes and molecular traits of CRKP strains isolated from pediatric patients and to investigate the prevalence of CRKP strains carrying hypervirulence-associated genes in pediatric clinical settings.

## Materials and Methods

### Strain Isolation and Antimicrobial Susceptibility Testing

KP strains were isolated from pediatric patients who were admitted to Henan Provincial Children's Hospital, the largest tertiary teaching children's hospital located in Central China. A total of 242 KP strains were collected between January 2021 and May 2023. After excluding 37 duplicate isolates recovered from the same patient or lacking antimicrobial susceptibility results, 205 KP strains were included in this study.

Antimicrobial susceptibility testing was performed in hospital microbiology laboratories via the minimum inhibitory concentration (MIC) method or the Kirby–Bauer disk diffusion method, with *Escherichia coli* ATCC 25922 serving as the quality control strain. The results were interpreted according to the breakpoints defined by the Clinical and Laboratory Standards Institute (CLSI) standards [25]. Interpretive criteria for tigecycline and polymyxin B followed the breakpoints established by the European Committee on Antimicrobial Susceptibility Testing (EUCAST) [26].

Multidrug-resistant (MDR) strains were defined as isolates harboring antimicrobial resistance genes from at least three different antimicrobial classes. CRKP strains were defined as those exhibiting MICs ≥ 4 μg/mL for meropenem or imipenem [25].

### Clinical Data and Patient Classification

Clinical data were obtained from the electronic medical records. All patient data were collected via a structured case report form, which recorded demographics, underlying diseases, ward unit, discharge diagnosis, length of hospitalization, admission date, clinical presentation, intravascular catheter use, mechanical ventilation, inflammatory biomarkers, imaging findings, and clinical outcomes. Underlying diseases in pediatric patients were categorized as prematurity, congenital heart disease, metabolic diseases, hematologic malignancies, immunodeficiencies and congenital anomalies. Patients were stratified by age at admission into 5 groups: 0–28 days, 29 days–2 months, 3–11 months, 1–5 years, and > 5 years. Neonates with low birth weights were further classified as follows: low birth weight (LBW, 1,500 g ≤ birth weight [BW] < 2,500 g), very low birth weight (VLBW, 1,000 g ≤ BW < 1,500 g), and extremely low birth weight (ELBW, BW < 1,000 g).

A poor outcome was defined as in-hospital death or “hopeless discharge”. Hopeless discharge referred to pediatric patients discharged at their parents’ request because their condition was incurable and further medical intervention was deemed futile.

### Whole-Genome Sequencing and Bioinformatic Analysis

All KP isolates were cultured overnight on lysogeny broth agar plates. Total DNA was purified and extracted via the magnetic particle method. Indexed DNA libraries were prepared via the use of the Qubit™ dsDNA HS Assay Kit (Thermo Fisher, USA). Sequencing was conducted on the Illumina NovaSeq 6000 platform via the Hieff NGS® MaxUp II DNA Library Prep Kit (Yeasen, China). The draft genomes of each isolate were assembled into contigs via SPAdes (version 3.15.5) [27], and the resulting contigs were filtered for a sequencing depth ≥ 10 bp and a scaffold length ≥ 300 bp. The sequence type (ST), antibiotic resistance genes (ARGs), virulence genes (*iuc*, *rmpA2*, etc.), virulence scores, capsule (K) serotypes, and O antigen of lipopolysaccharide (LPS) serotypes were predicted via KleBorate [28]. Plasmid types and replicons were identified in genomic assemblies via PlasmidFinder [29]. Single-nucleotide polymorphisms (SNPs) were identified via Snippy [30], and core genome SNPs differential analysis was performed via the R package vcfR (version 1.15.0) [31]. Core genome SNPs were used to perform phylogenetic analysis via the maximum likelihood method. The phylogenetic tree was generated with RAxML using strain HS11286 (PRJNA84387) as the reference genome and visualized with iTOL (version 6.9.1) [32]. The average nucleotide identity (ANI) was calculated via FastANI [33]. Principal component analysis (PCA) was performed via genome-wide complex trait analysis (GCTA) [34]. The population structure was analyzed via ADMIXTURE [35].

Genomic data from 188 CRKP strains isolated from adult patients in Henan province have been previously described [36].

### Statistical Analysis

The data were entered and initially processed via Excel 2024 (Microsoft) and further analyzed via SPSS software (IBM Statistics version 20.0). The results are expressed as absolute numbers and percentages or as medians and interquartile ranges (IQR). Categorical variables were compared via the chi-square test or Fisher’s exact test, whereas continuous variables were compared via Student’s *t* test or the Mann‒Whitney *U* test, depending on the data distribution. A *P* value < 0.05 was considered statistically significant. LASSO regression analysis was employed to identify potential risk factors, and multivariate binomial regression analysis was conducted to evaluate independent risk factors associated with *iuc*^+^*rmpA2*^+^ CRKP isolation. The results are presented as *P* values, odds ratios (ORs), and 95% confidence intervals (CIs). Data analyses and visualizations were performed via OriginPro 2024 and R Studio.

## Results

### Characteristics of Pediatric Patients and the Distribution of KP Isolates

A total of 205 pediatric patients with KP isolates were included. Among these patients, whose median age was 3.1 months (IQR: 1.1–7.5 months), 129 (62.9%) were male. The predominant age groups were 3–11 months (33.2%, n = 68) and 29 days–2 months (26.8%, n = 55). Sixty-seven patients (32.7%) were premature and had LBW. One hundred and forty-two patients (69.3%) were from general medical wards, 28 (13.7%) were from the pediatric intensive care unit (PICU), and 16 (7.8%) were from the neonatal intensive care unit (NICU). Ninety-four patients (45.9%) underwent invasive procedures (Table 1). Four patients (2.0%) died during hospitalization, and seven (3.4%) were discharged hopelessly at their parents’ request.

**Table 1:** Characteristics of the children according to the pattern of Carbapenem resistance

Variable	Total	CSKP (n = 118, %)	CRKP (n = 87, %)	*P*
Sex				
Male	129 (62.9)	76 (64.4)	53 (60.9)	0.72
Female	76 (37.1)	42 (35.6)	34 (39.1)	0.72
Age (months)	3.1 (1.1, 7.5)	4.2 (2, 34)	1.7 (0.7, 4.2)	**<0.001**
0–28 days	44 (21.5)	16 (13.6)	28 (32.2)	**<0.001**
29 days–2 months	55 (26.8)	30 (25.4)	25 (28.7)	0.71
3–11 months	68 (33.2)	38 (32.2)	30 (34.5)	0.85
1–5 years	18 (8.8)	16 (13.6)	2 (2.3)	**0.01**
>5 years	20 (9.8)	18 (15.3)	2 (2.3)	**<0.001**
BW				
Full-term	138 (67.3)	97 (82.2)	41 (47.1)	**<0.001**
LBW	24 (11.7)	9 (7.6)	15 (17.2)	0.06
VLBW	38 (18.5)	11 (9.3)	27 (31.0)	**<0.001**
ELBW	5 (2.4)	1 (0.8)	4 (4.6)	0.17
Specimen				
Sputum	159 (77.6)	82 (69.5)	77 (88.5)	**<0.001**
Blood	7 (3.4)	6 (5.1)	1 (1.1)	0.24
Cerebrospinal fluid	2 (1)	2 (1.7)	0	0.51
Bronchoalveolar lavage fluid	7 (3.4)	4 (3.4)	3 (3.4)	1.00
Urine	11 (5.4)	8 (6.8)	3 (3.4)	0.46
Others	19 (9.3)	16 (13.6)	3 (3.4)	**0.03**
Underlying disease				
Prematurity	67 (32.7)	19 (16.1)	48 (55.2)	**<0.001**
Congenital heart disease	27 (13.2)	10 (8.5)	17 (19.5)	**0.04**
Congenital anomalies	25 (12.2)	14 (11.9)	11 (12.6)	1.00
Metabolic diseases	13 (6.3)	7 (5.9)	6 (6.9)	1.00
Hematologic malignancies	5 (2.4)	5 (4.2)	0	0.07
Immunodeficiencies	3 (1.5)	0	3 (3.4)	0.08
Others	17 (8.3)	10 (8.5)	7 (8)	1.00
Departments				
General medical ward	142 (69.3)	81 (68.6)	61 (70.1)	0.94
NICU	16 (7.8)	6 (5.1)	10 (11.5)	0.15
PICU	28 (13.7)	14 (11.9)	14 (16.1)	0.51
Surgical ward	15 (7.3)	13 (11)	2 (2.3)	0.04
Hematology-oncology ward	4 (2)	4 (3.4)	0	0.14
Invasive procedures and devices	94 (45.9)			
PICC	14 (6.8)	8 (6.8)	6 (6.9)	1.00
Nasogastric tube	83 (40.5)	28 (23.7)	55 (63.2)	**<0.001**
Invasive mechanical ventilation	32 (15.6)	13 (11)	19 (21.8)	0.06
Surgical operation	10 (4.9)	5 (4.2)	5 (5.7)	0.87
Mortality or hopeless discharge	11 (5.4)	3 (2.5)	8 (9.2)	0.08

*CSKP* carbapenem-sensitive *Klebsiella pneumoniae*, *CRKP* carbapenem-resistant *Klebsiella pneumoniae*, *BW* birth weight, *LBW* low birth weight, *VLBW* very low birth weight, *ELBW* extremely low birth weight, *PICC* peripherally inserted central catheter, *NICU* neonatal intensive care unit, *PICU* pediatric intensive care unit

Significant *P* values (<0.05) are highlighted in bold

Among the 205 KP isolates, 159 (77.6%) were obtained from sputum, 11 (5.4%) from urine, and 7 (3.4%) each from blood and bronchoalveolar lavage fluid. Among these isolates, 87 (42.4%) were CRKP, and 118 (57.6%) were CSKP. On the basis of the presence of the *rmpA2* and *iuc* genes, the isolates were categorized as either classical KPs or *iuc*^+^*rmpA2*^+^ KPs. Among the CRKP isolates, 32 (36.8%) were classical CRKP, and 55 (63.2%) were *iuc*^+^*rmpA2*^+^ CRKP. Among the CSKP isolates, 91 (77.1%) were classical CSKPs, and 27 (22.9%) were *iuc*^+^*rmpA2*^+^ KPs (Table S1).

Compared with patients with CSKP isolates, those with CRKP isolates were younger (median age: 1.7 vs. 4.2 months, *P* < 0.001) and presented significantly greater proportions of VLBW (31.0% vs. 9.3%, *P* < 0.05), prematurity (55.2% vs. 16.1%, *P* < 0.05), and congenital heart disease (19.5% vs. 8.5%, *P* < 0.05). Additionally, the proportion of patients who received nasogastric tube insertion was significantly greater in the CRKP group (63.2% vs. 23.7%, *P* < 0.001) (Table 1). No statistically significant differences in clinical characteristics were detected between classical CRKP and *iuc*^+^*rmpA2*^+^ CRKP isolates (Table S1).

### Antimicrobial Susceptibility Patterns of the Pediatric CRKP and ***iuc***^+^***rmpA2***^+^ CRKP Isolates

As shown in Table 2, both classical CRKP and *iuc*^+^*rmpA2*^+^ CRKP isolates presented extremely high resistance rates (> 96%) to commonly used antibiotics, including cefepime, ceftazidime, aztreonam, and piperacillin/tazobactam. Additionally, *iuc*^+^*rmpA2*^+^ CRKP strains presented significantly higher resistance rates than classical CRKP strains to aztreonam (100% vs. 90.6%, *P* < 0.05), amikacin (67.3% vs. 40.6%, *P* < 0.05), and levofloxacin (98.2% vs. 78.1%, *P* < 0.05). Conversely, the *iuc*^+^*rmpA2*^+^ KP isolates presented lower antibiotic resistance rates than the classical CSKP isolates against most of the antibiotics tested. Besides, CSKP isolates exhibited lower antibiotic resistance to most tested antibiotics compared with CRKP isolates (Table S2).

**Table 2:** Resistance patterns of *Klebsiella pneumoniae* (KP)

Antimicrobial drugs	Percentage (%) of resistance						
	Total	CSKP		*P*^a^	CRKP		*P*^b^
		Classical CSKP (n = 91)	*iuc*^+^*rmpA2*^+^ CSKP (n = 27)		Classical CRKP (n = 32)	*iuc*^+^*rmpA2*^+^ CRKP (n = 55)	
Piperacillin	151 (73.7)	58 (63.7)	7 (25.9)	**<0.001**	32 (100)	54 (98.2)	1.00
Piperacillin/Tazobactam	113 (55.1)	22 (24.2)	6 (22.2)	1.00	31 (96.9)	54 (98.2)	1.00
Cefepime	127 (62)	37 (40.7)	5 (18.5)	0.06	31 (96.9)	54 (98.2)	1.00
Ceftazidime	122 (59.5)	32 (35.2)	5 (18.5)	0.16	31 (96.9)	54 (98.2)	1.00
Cefuroxime	152 (74.1)	59 (64.8)	7 (25.9)	**<0.001**	31 (96.9)	55 (100)	0.37
Aztreonam	123 (60)	33 (36.3)	6 (22.2)	0.26	29 (90.6)	55 (100)	**0.05**
Amikacin	58 (28.3)	5 (5.5)	3 (11.1)	0.56	13 (40.6)	37 (67.3)	**0.03**
Ciprofloxacin	130 (63.4)	44 (48.4)	6 (22.2)	**0.03**	27 (84.4)	53 (96.4)	0.10
Levofloxacin	110 (53.7)	25 (27.5)	6 (22.2)	0.77	25 (78.1)	54 (98.2)	**0.01**
Imipenem	87 (42.4)	0	0	NT	32 (100)	55 (100)	NT
Meropenem	85 (41.5)	0	0	NT	32 (100)	55 (100)	NT
Trimethoprim/Sulfamethoxazole	41 (20)	33 (36.3)	4 (14.8)	0.06	3 (9.4)	1 (1.8)	0.14
Tigecycline	4 (2)	3 (3.3)	0	1.00	1 (3.1)	0	0.37

^a^The *P* value shows the significant difference between classical CSKP and *iuc*^+^*rmpA2*^+^ CSKP

^b^The *P* value shows the significant difference between classical CRKP and *iuc*^+^*rmpA2*^+^ CRKP

*NT* not test

Significant *P* values (<0.05) are highlighted in bold

### Predominance of Pediatric ST11–KL47–KPC2 KP Isolates with a High Prevalence of Virulence Genes

**Fig. 1:**
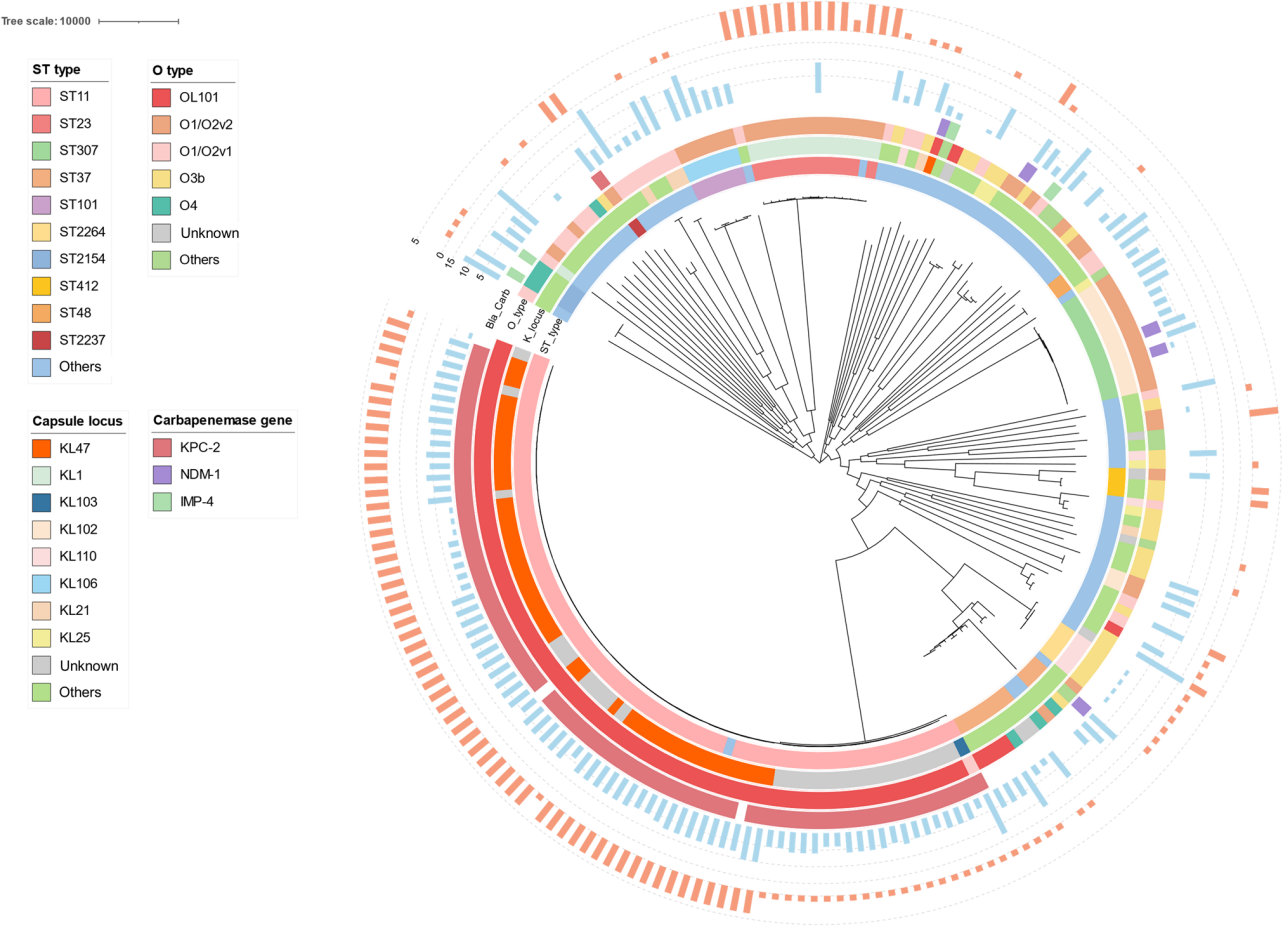
The phylogeny of 205 pediatric *Klebsiella pneumoniae* (KP) isolates. A reference ST11–KL103–KPC2 carbapenem-resistant KP (CRKP) HS11286 was added to the maximum likelihood tree. From inside to outside, information on the STs, capsule locus O types, and carbapenemases is provided by each track. In the bar chart, the inner bar chart displays the number of antimicrobial resistance (AMR) genes for each strain, and the outermost bar chart displays the virulence score for each strain

The phylogeny of 205 pediatric KP isolates is shown in Fig. 1. Among the 87 CRKP isolates, a total of 10 STs were identified, with ST11 (87.4%) being the most prevalent. With respect to capsule types, KL47 (55.2%) predominated. Among the 118 CSKP isolates, 69 STs were detected, of which ST23 (11.0%), ST307 (7.6%), ST37 (7.6%), and ST101 (5.1%) were relatively common. The predominant capsule types were KL1 (12.7%), KL102 (9.3%), KL110 (5.9%), and KL106 (5.1%) (Fig. 1).

Among the 87 CRKP isolates, KPC-2 (89.7%) was the predominant carbapenemase, followed by NDM-1 (5.7%) and IMP-4 (4.6%). Seventy-four (85.1%) CRKP strains harbored 4–10 AMR genes, and 54 (62.1%) CRKP isolates harbored ESBL genes, with CTX-M-65 (72.2%) being the most common (Fig. S1). Moreover, KL47 (55.2%) was the predominant capsule type, and OL101 (88.5%) was the most common O-antigen type. Among the 79 ST11 KP isolates, 76 (96.2%) carried KPC-2, and 48 (60.8%) belonged to KL47. The ST11–KL47–KPC2 genotype (52.9%) was the predominant genotype among the CRKP isolates (Fig. S1).

**Fig. 2:**
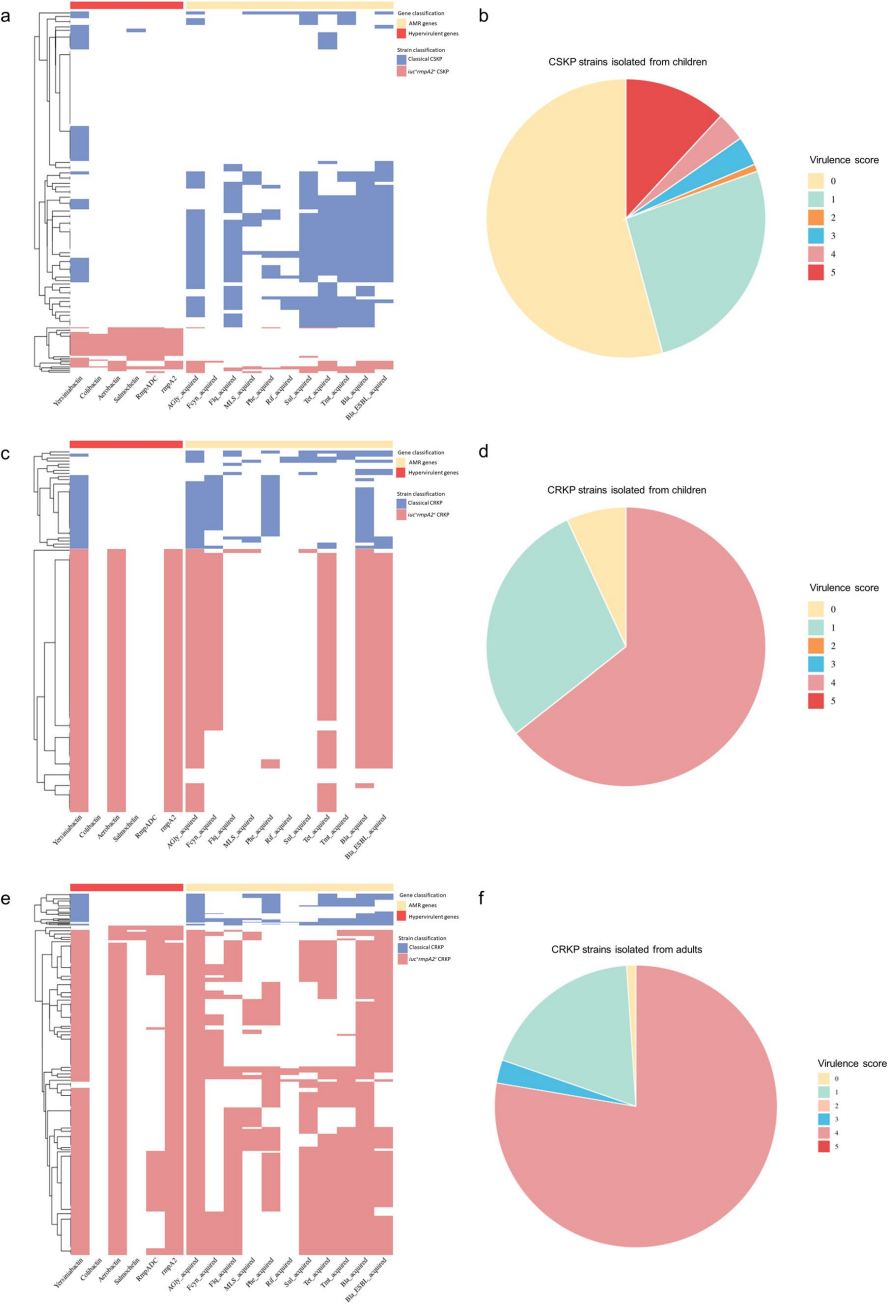
Distribution of virulence and AMR profiles in pediatric carbapenem-sensitive KP (CSKP) isolates, CRKP isolates and adult CRKP isolates. **a**, **c**, **e** The heatmap illustrating the distribution of acquired AMR genes and virulence genes in pediatric CSKP, CRKP, and adult CRKP isolates, respectively. **b**, **d**, **f** The pie charts showing the proportions of isolates with different virulence scores: pediatric CSKP, CRKP, and adult CRKP isolates, respectively

Compared with the 205 pediatric KP isolates, the CRKP isolates presented significantly greater frequencies of virulence genes, including *ybt* (92.0% vs. 43.2%, *P* < 0.01), *iuc* (63.2% vs. 19.5%, *P* < 0.01), and *rmpA2* (63.2% vs. 17.8%, *P* < 0.01), than did the CSKP isolates. CRKP isolates also exhibited significantly higher virulence scores (median score 4 vs. 0, *P* < 0.01) (Fig. 2a–d).

### Comparative Genomic Epidemiology of Pediatric and Adult CRKP Isolates in Henan Province

**Fig. 3:**
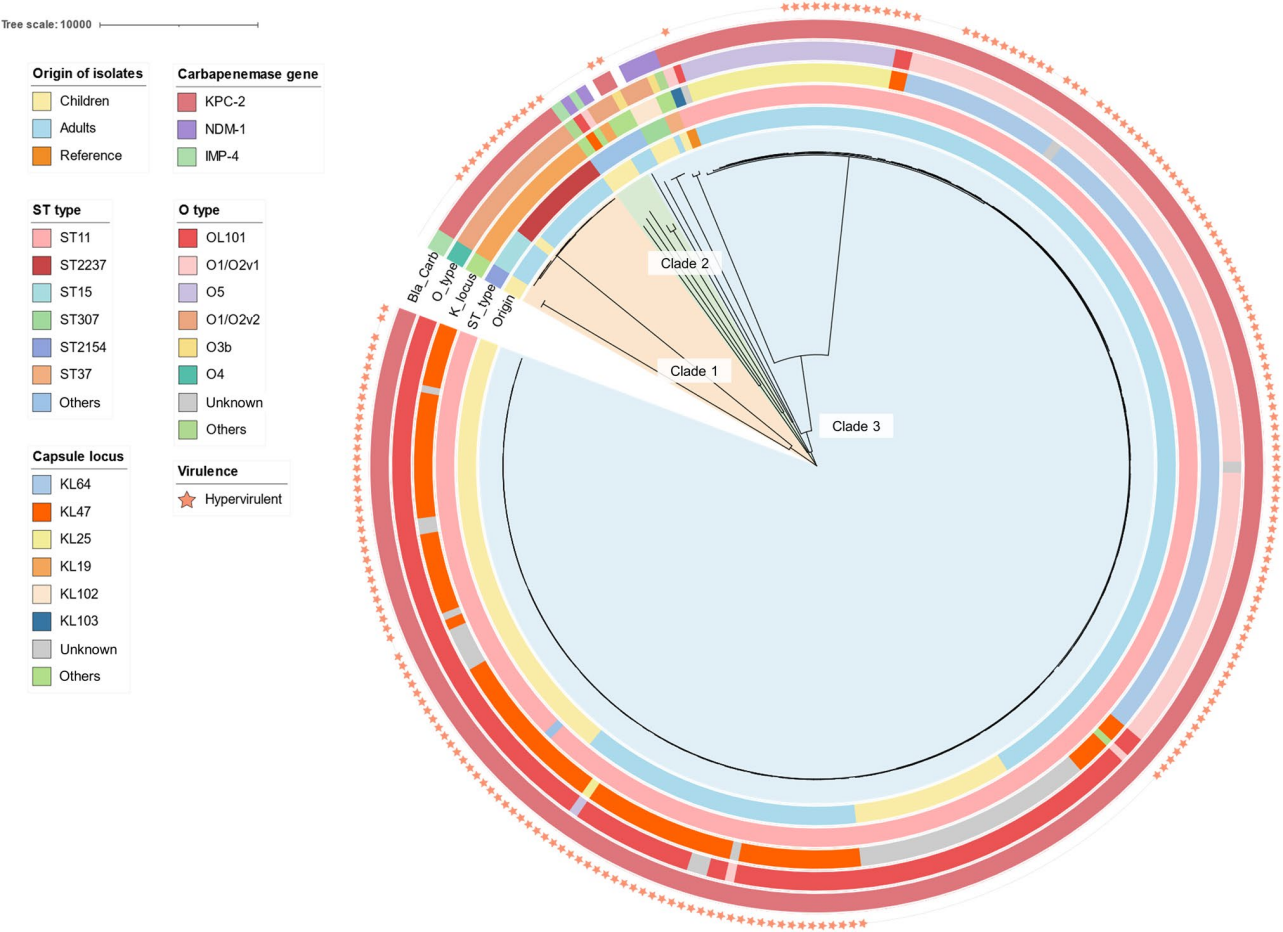
The phylogeny of 87 pediatric CRKP isolates and 188 adult CRKP isolates. A reference ST11–KL103–KPC2 CRKP HS11286 was added to the maximum likelihood tree. From the inside to the outside, information on the origin, ST type, capsule locus O type, and carbapenemase activity is provided by each track. The outermost stars indicate *iuc*^+^*rmpA2*^+^ CRKP isolates

To characterize regional epidemiology and compare molecular profiles between pediatric and adult CRKP isolates, we integrated genomic data from 188 adult CRKP isolates previously reported in Henan province [36]. The phylogenies of the pediatric (n = 87) and adult (n = 188) CRKP isolates are presented in Fig. 3. ST11 was the predominant ST among both pediatric (86.2%, 75/87) and adult (89.3%, 168/188) CRKP isolates. Almost all the ST11 CRKP isolates clustered in clade 3 of the phylogenetic tree, indicating close genomic relatedness with minimal SNPs differences. ST2237 was more prevalent in adults (2.7%, 5/188) than in pediatric isolates (1.1%, 1/87), whereas ST15 was detected exclusively in adults (2.7%, 5/188). Apart from one isolate carrying *bla*_NDM-1_, all the other adult CRKP isolates harbored the *bla*_KPC-2_ gene.

With respect to capsule loci, KL64 (48.4%, 91/188) was the predominant type in adults but was absent in pediatric isolates, whereas KL47 was more prevalent in pediatric (55.2%, 48/87) than in adult isolates (22.3%, 42/188).

Furthermore, 152 adult strains (80.9%) were *iuc*^+^*rmpA2*^+^ CRKP, which was significantly greater than the prevalence among pediatric isolates (63.2%). Additionally, adult *iuc*^+^*rmpA2*^+^ CRKP isolates harbored the *rmpADC* gene more frequently and presented higher virulence scores (Fig. 2e, f). Moreover, 150 adult isolates (79.8%) were *iuc*^+^*rmpA2*^+^ MDR strains, which was also significantly greater than the number of pediatric isolates (59.8%).

For plasmid replicons, IncFII (pHN7A8) (91.4%) and IncHIB (63.2%) predominated among adult *iuc*^+^*rmpA2*^+^ CRKP isolates, whereas IncFII (pHN7A8) (98.2%), IncFIB(K) (49.1%), and IncHIB (34.5%) were most prevalent in pediatric *iuc*^+^*rmpA2*^+^ CRKP isolates (Fig. S2).

### Pediatric and Adult CRKP Strains Demonstrate Genetic Similarity

Among 87 pediatric CRKP isolates, 46 (52.9%) ST11–KL47–KPC2 CRKP strains presented high genetic similarity (< 30 SNPs, ANI > 99.5%) (Tables S3, S5). Additionally, 24 ST11–KL47–KPC2 CRKP strain isolates positive for *iuc* and *rmpA2* presented fewer than 10 SNPs, indicating potential intrahospital clonal transmission and evolution of *iuc*^+^*rmpA2*^+^ CRKP strains during the study period. Similarly, several ST11–KL64, ST15–KL19, and ST2237–KL19 CRKP isolates presented fewer than 10 SNPs (Table S4). In addition, 157 (83.5%) ST11–KL64/KL47/KL25–KPC2 strains and 11 (5.6%) ST11–KL19–KPC2 isolates presented high genomic similarity (ANI > 99.5%) (Table S6).

Moreover, several pediatric and adult ST11–KL47–KPC2 strains presented high genomic relatedness (ANI > 99.8%) and fewer than 10 SNPs, suggesting clonal transmission of CRKP between pediatric and adult patients in Henan province. Additionally, compared with adult ST2237 CRKP strains, the single pediatric ST2237–CRKP isolate presented greater than 99.9% ANI similarity and fewer than 30 SNPs. Several pediatric and adult ST11–KL47–KPC2 isolates also presented fewer than 30 SNPs (Table S4). These findings indicated that these strains may have originated from a common ancestor, disseminated among different populations, and continued evolving.

**Fig. 4:**
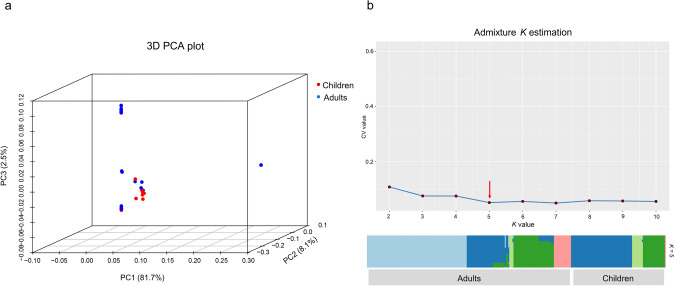
Population genetic analysis of CRKP strains isolated from pediatric patients and adults. **a** Three-dimensional principal component analysis (PCA) plot displaying the genetic subgroups of CRKP isolates on the basis of SNPs profiles in child (red) and adult (blue) populations. The axes represent the top three principal components (PCs) (PC1–3), which explain 81.7%, 8.1%, and 2.5% of the total genomic variance, respectively. **b** The line plot shows the cross-validation (CV) error values for different tested *K* values (*K* represents the number of putative clusters in the population structure analysis). The red arrow marks the optimal *K* value (*K* = 5) with minimal CV error. In the population structure analysis of the CRKP isolates, the vertical column represents the adult and child populations, and the colored segments within the column indicate proportional ancestry contributions from the *K* = 5 putative ancestral populations

The genetic subgroups of CRKP isolates from children (red) and adults (blue) are shown in the principal component analysis (PCA) plot (Fig. 4a). All CRKP isolates clustered into three groups, each containing closely related pediatric and adult isolates exhibiting minor SNPs differences (Fig. S3, Table S7). Admixture analysis (optimal *K* value = 5) revealed three predominant ancestries shared between pediatric and adult CRKP isolates. (Fig. 4b, S4).

### Risk Factors Associated with Pediatric ***iuc***^+^***rmpA2***^+^ CRKP Isolation

**Fig. 5:**
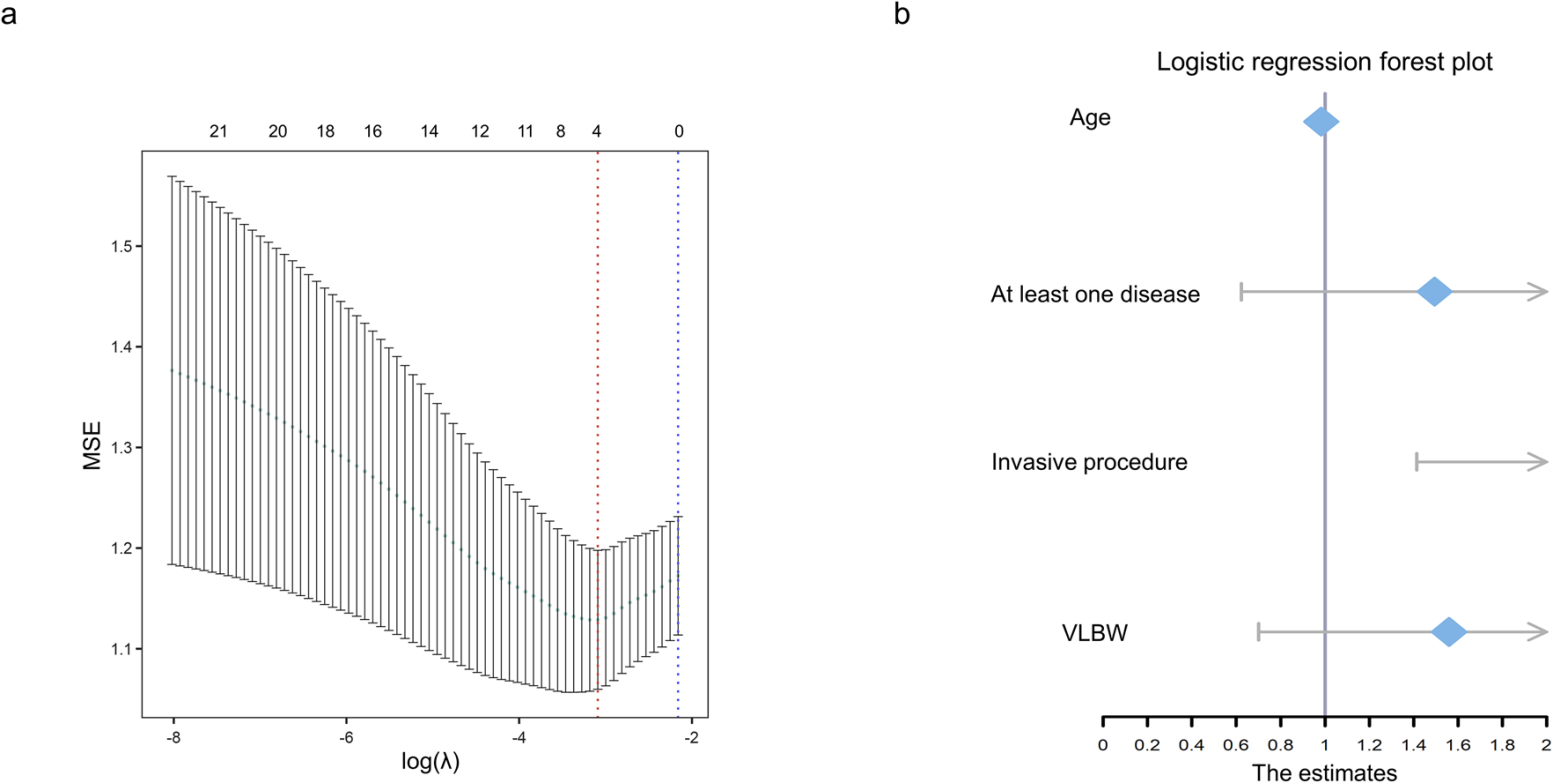
Regression analysis of risk factors associated with *iuc*^+^*rmpA2*^+^ CRKP isolation. **a** The plot shows the cross-validated mean squared error (MSE) across a range of log(λ) penalty values. The optimal regularization parameter (λ) was four (marked by a red dotted line). **b** The forest plot displays odds ratios (ORs) with 95% confidence intervals (CIs) for four risk factors associated with *iuc*^+^*rmpA2*^+^ CRKP isolation, identified by multivariate binomial regression. The reference line at OR = 1 indicates no association

Influencing factors associated with pediatric *iuc*^+^*rmpA2*^+^ CRKP isolation were analyzed among 205 pediatric KP strains. Initially, 22 influencing factors were screened, and four potential risk factors for pediatric *iuc*^+^*rmpA2*^+^ CRKP isolation were identified via LASSO regression at the optimal regularization parameter (λ = 4) (Table S8, Fig. S5). Subsequent multivariate binomial regression analysis revealed that older age was a protective factor (*P* < 0.05; OR: 0.984; 95% CI: 0.967–0.997), whereas undergoing invasive procedures was a significant risk factor (*P* < 0.05; OR: 2.917; 95% CI: 1.414–6.205) (Table 3, Fig. 5).

**Table 3:** Multiple regression analysis of risk factors associated with *iuc*^+^*rmpA2*^+^ CRKP isolation

Variable	Total	*iuc*^+^*rmpA2*^+^ CRKP (n = 55)	Other KP (n = 150)	*P*	Multivariate analysis		
					*P*	OR	95% CI
Age (months)	3.1 (1.1,7.5)	2.3 (0.9, 5.4)	3.4 (1.5, 9.7)	0.018	**0.039**	0.984	0.967–0.997
VLBW	67 (32.7)	26 (47.3)	41 (27.3)	0.008	0.28	1.56	0.700**–**3.477
At least one underlying disease	129 (62.9)	44 (80.0)	85 (56.7)	0.003	0.371	1.495	0.623**–**3.676
Invasive procedures^†^	94 (45.9)	37 (67.3)	57 (38.0)	0.000	**0.004**	2.917	1.414–6.205

^†^Invasive procedures involve any of the following: peripherally inserted central catheters, nasogastric tubes, invasive mechanical ventilation, and surgical operations

Significant *P* values (<0.05) from the multiple regression analysis are highlighted in bold

## Discussion

This study revealed a high prevalence (63.2%) of hypervirulence-associated genes among pediatric CRKP isolates, and these strains displayed clonal transmission. ST11–KL47 (52.9%) CRKP was identified as the predominant genotype among the pediatric CRKP isolates, whereas ST11–KL64 CRKP was not detected. By integrating genomic data from local adult CRKP isolates, we identified close genetic relatedness between pediatric and adult CRKP isolates. These findings suggest that the strains share a common ancestor and have disseminated between general and pediatric hospitals. Additionally, older age was identified as a protective factor, and invasive medical procedures represented a clinical risk factor associated with pediatric *iuc*^+^*rmpA2*^+^ CRKP isolation. Our results emphasize the genetic correlation between pediatric and adult CRKP strains and underscore the extensive dissemination of hypervirulence-associated genes among pediatric CRKP strains.

Carbapenemase production represents the primary molecular mechanism underlying CRKP emergence. In this study, KPC-2 (89.7%) was the predominant carbapenemase among the pediatric CRKP isolates, which is consistent with the findings for adult CRKP isolates (99.4%) in Henan province [36]. However, previous surveillance studies reported distinct carbapenemase profiles between pediatric and adult CRKP isolates. KPC has been reported predominantly among adult patients, accounting for 93.6% in China, 68.8% in South America, and 80.6% in the United States [37]. In contrast, NDM has frequently been identified as the most common carbapenemase among pediatric patients, with prevalence rates of 20% in Iran and 49.0% in China [7, 38]. Previous studies reported both clonal and horizontal transmission of NDM-producing CRKP isolates among pediatric patients [39], whereas our study revealed the transmission of KPC-producing CRKP isolates between pediatric patients and local adult patients. Therefore, regional surveillance of carbapenemase production is necessary to optimize clinical therapy. Given the high prevalence of KPC carbapenemase production, recently introduced antibiotics such as ceftazidime-avibactam remain effective therapeutic options for CRKP infections in Henan province [4].

To our knowledge, this study is the first to report a notably high prevalence (63.2%) of *iuc*^+^*rmpA2*^+^ CRKP among pediatric patients identified through whole-genome sequencing. The prevalence of this genotype among adult patients in the same region was even greater (80.9%) [36]. Previous surveillance data revealed an increase in CRKP strains harboring hypervirulence-associated genes among adults in China, with regional variations ranging from 5.4% to 52.7% [40, 41]. However, studies investigating these genes in pediatric populations are limited [24, 42]. Compared with classical hvKPs, CRKP strains carrying hypervirulence-associated genes exhibit increased transmissibility and greater resistance [40]. With the spread of these high-risk strains, surveillance efforts targeting hypervirulence-associated genes in pediatric CRKP isolates are necessary to characterize the epidemiological trends and raise clinical awareness.

In this study, ST11–KL47–KPC2 was the predominant genotype among the pediatric CRKP isolates, accounting for 52.9% of the strains carrying hypervirulence-associated genes. In contrast, ST11–KL64 strains have been identified as the predominant hv-CRKP clone among adult patients in China [43]. These strains emerged from an ancestral ST11–KL47-like lineage approximately 2011, following a capsular locus replacement from KL47 to KL64 [44, 45]. Genomic surveillance data (2013–2017) among adults in China revealed that ST11–KL47 was the predominant subclone prior to 2015 [15]. Previous studies reported that ST11–KL47 isolates carrying hypervirulence-associated genes exhibit lower pathogenicity in adults [46], which is consistent with our findings. Our results further indicated that ST11–KL47 CRKP strains with hypervirulence-associated genes were emerging and evolving, demonstrating a high prevalence but relatively low pathogenicity. Compared with the KL47 strains, the ST11–KL64 isolates exhibit greater pathogenicity [9, 47]. Clinical studies reported significantly higher rates of septic shock and mortality in patients with bloodstream infections caused by the ST11–KL64–CRKP strains [44].

Furthermore, the ST11–KL64 CRKP strains exhibit increased recombination-driven genetic variation and carry more mobile genetic elements, facilitating virulence evolution and interhospital transmission [9]. Thus, ST11–KL64 has been identified as a key genotype associated with hv-CRKP transmission. ST11–KL64 hv-CRKP strains evolved primarily from ST11–KL64 CRKP through the acquisition of the pLVPK-like virulence plasmid from hvKP [48, 49]. However, the high-risk ST11–KL64 subclone was not identified in this study, despite its high prevalence (48.4%) among local adult CRKP isolates. Nevertheless, our genomic analysis revealed close genetic relationships between pediatric and adult CRKP isolates. Additionally, SNPs analysis suggested some clonal transmission among the isolates. Therefore, it is imperative to strengthen genomic surveillance to detect capsule-type shifts among pediatric CRKP strains and to remain vigilant against the emergence and dissemination of the high-risk ST11–KL64 subclone in pediatric patients.

We identified two factors influencing the isolation of pediatric CRKP strains with hypervirulence-associated genes: older age as a protective factor and invasive medical procedures as a clinical risk factor. Investigations into the risk factors for infection with these high-risk strains remain limited and ongoing. Previous adult-focused studies provide reference data: a single-center study in Zhejiang identified recent antibiotic therapy and invasive procedures as significant risk factors [50]. Another study in Fujian identified bed transfers as a risk factor for hv-CRKP infection [51]. Additionally, a study in Nanjing reported tracheotomy as a risk factor for hv-CRKP-related intra-abdominal infections [52]. These adult data suggest that risk factors may be shared across age groups and highlight the need for further high-quality studies among pediatric patients. Although the ST11–KL47 *iuc*^+^*rmpA2*^+^ CRKP isolates presented lower pathogenicity and appeared less associated with life-threatening infections in pediatric patients in our study, their emergence and colonization still represent a potential threat to children with immature immune systems, particularly neonates and preterm infants. Therefore, pediatric patients with risk factors for infection or colonization by CRKP carrying hypervirulence-associated genes require increased clinical attention and intensified targeted infection control measures.

This study has two main limitations. First, its single-center design covers a relatively narrow geographic area, limiting its representativeness across other locales. Second, the retrospective nature of the study may introduce information bias. Nevertheless, our findings provide valuable data on the prevalence of virulence genes among pediatric CRKP isolates in China and demonstrate genomic relatedness between pediatric and local adult CRKP strains. Further multicenter prospective studies are needed to comprehensively investigate the distribution of virulence genes and evolutionary trajectories in pediatric CRKP isolates.

## Conclusions

This study investigated the clinical and virulence traits of CRKP strains from pediatric patients. ST11–KL47 (52.9%) was identified as the predominant genotype, whereas the high-risk ST11–KL64 subclone was not detected. We found a high prevalence (63.2%) of clonal *iuc*^+^*rmpA2*^+^ CRKP strains and clonal transmission among pediatric patients. Additionally, this study revealed close genetic relatedness between pediatric and adult CRKP strains carrying hypervirulence-associated genes, suggesting that these strains may have originated from a common ancestor and evolved in distinct populations. Younger age and exposure to invasive procedures were identified as clinical risk factors for the isolation of CRKP strains carrying hypervirulence-associated genes in pediatric patients, warranting clinical vigilance.

## Supplementary Information

Below is the link to the electronic supplementary material.

Supplementary file1 (DOCX 1124 KB)

Supplementary file2 (XLSX 1013 KB)

## Data Availability

All genome sequences were deposited in the National Genomics Data Center under the Bioproject accession number PRJNA1171176.
