# Supplementary material for: Virulence and Genomic Profiles of Klebsiella pneumoniae Isolated from Pediatric Patients in Henan, China (2021–2023)
Source: Curr Med Sci. 2025 Nov 17;45(6):1391–403. doi: 10.1007/s11596-025-00137-w (PMC12748107; doi:10.1007/s11596-025-00137-w)
Supplement: Supplementary file 1 — Supplementary file1 (DOCX 1124 KB) [file 11596_2025_137_MOESM1_ESM.docx]

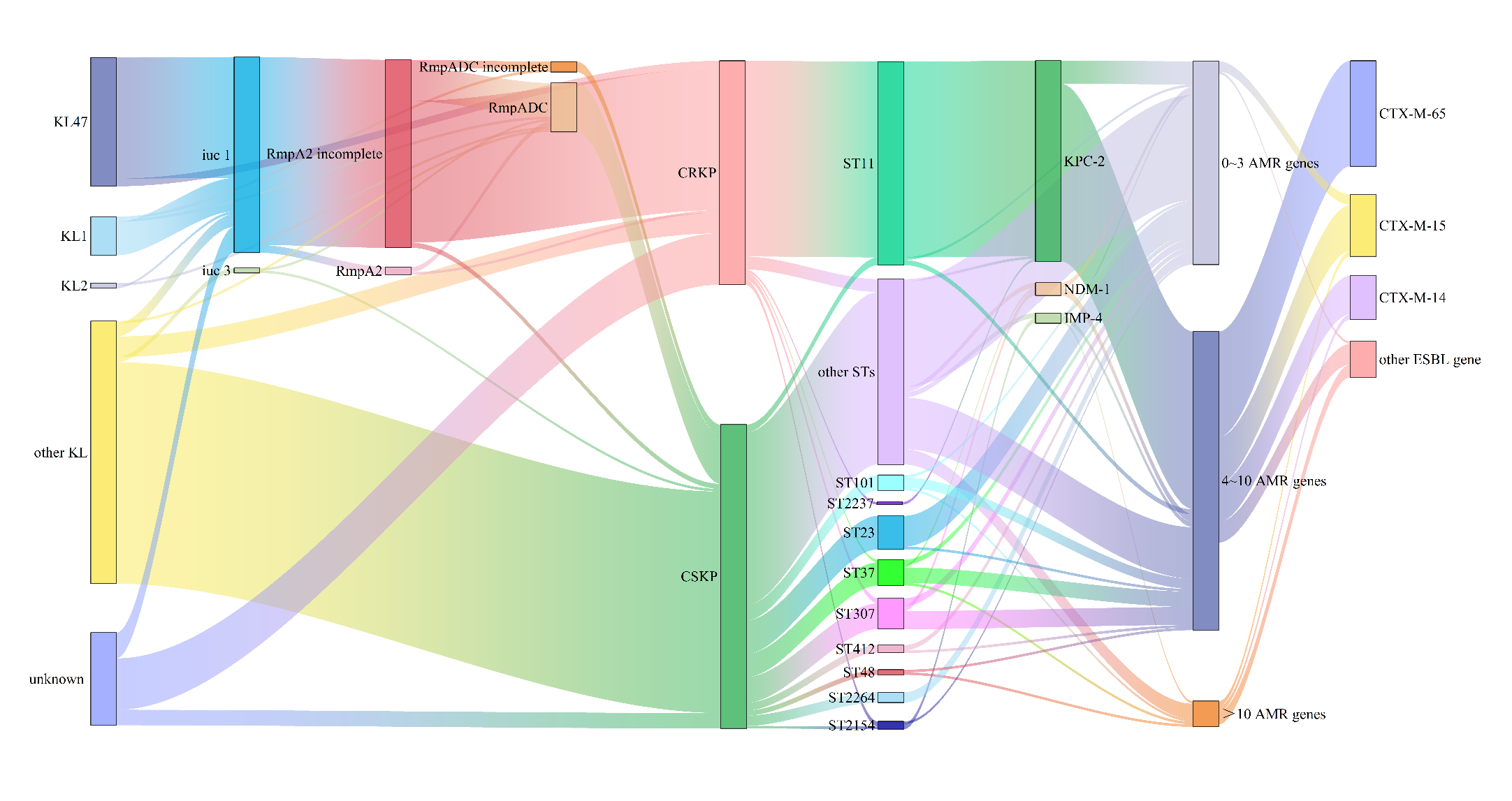


**Fig. S1** The correlation of genetic information in 205 KP isolates


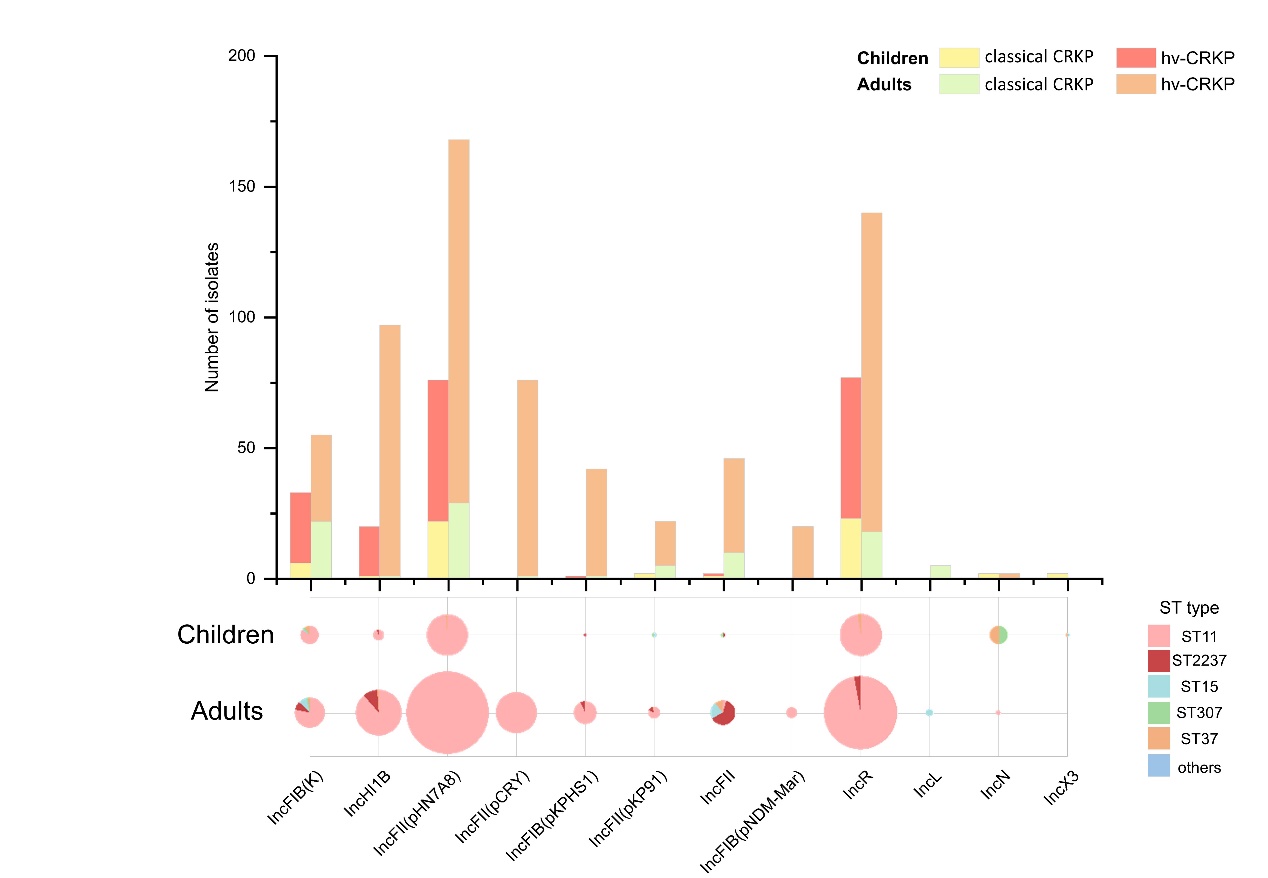


**Fig. S2** Molecular analysis of CRKP strains isolated from pediatric patients and adults. Bar chart displayed the strain counts harboring different plasmid replicons, stratified by common CRKP and *iuc*^+^*rmpA2*^+^ CRKP isolates and grouped by children and adult population. Pie chart representation of ST type proportions among CRKP strains harboring different replicons, and the pie size corresponded to the number of CRKP strains


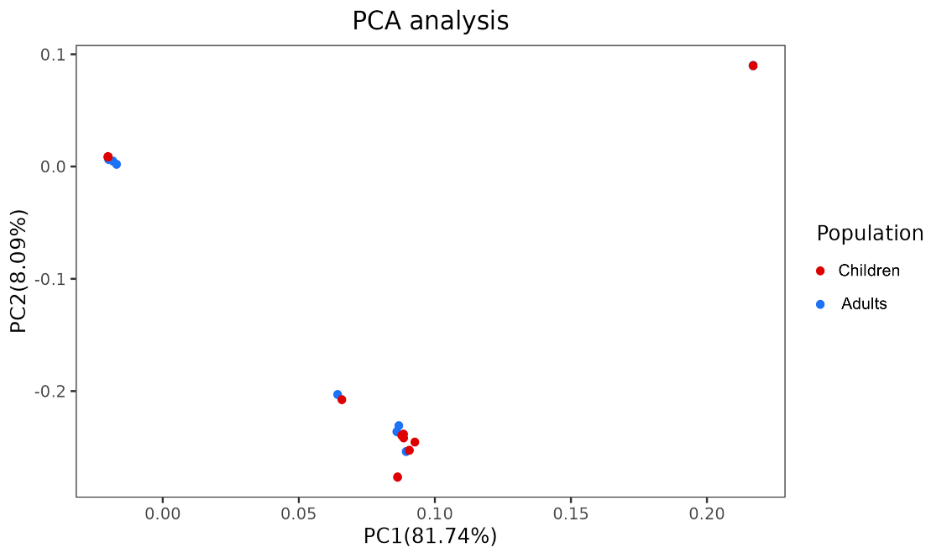


**Fig. S3** Population genetic analysis of CRKP strains isolated from pediatric patients and adults. The two-dimensional PCA plot displayed the genetic subgroups of CRKP isolates in children (red) and adults’ populations (blue). PC1 (X axes) and PC2 (Y axes) explaining 81.7% and 8.1% of total genomic variance, respectively.


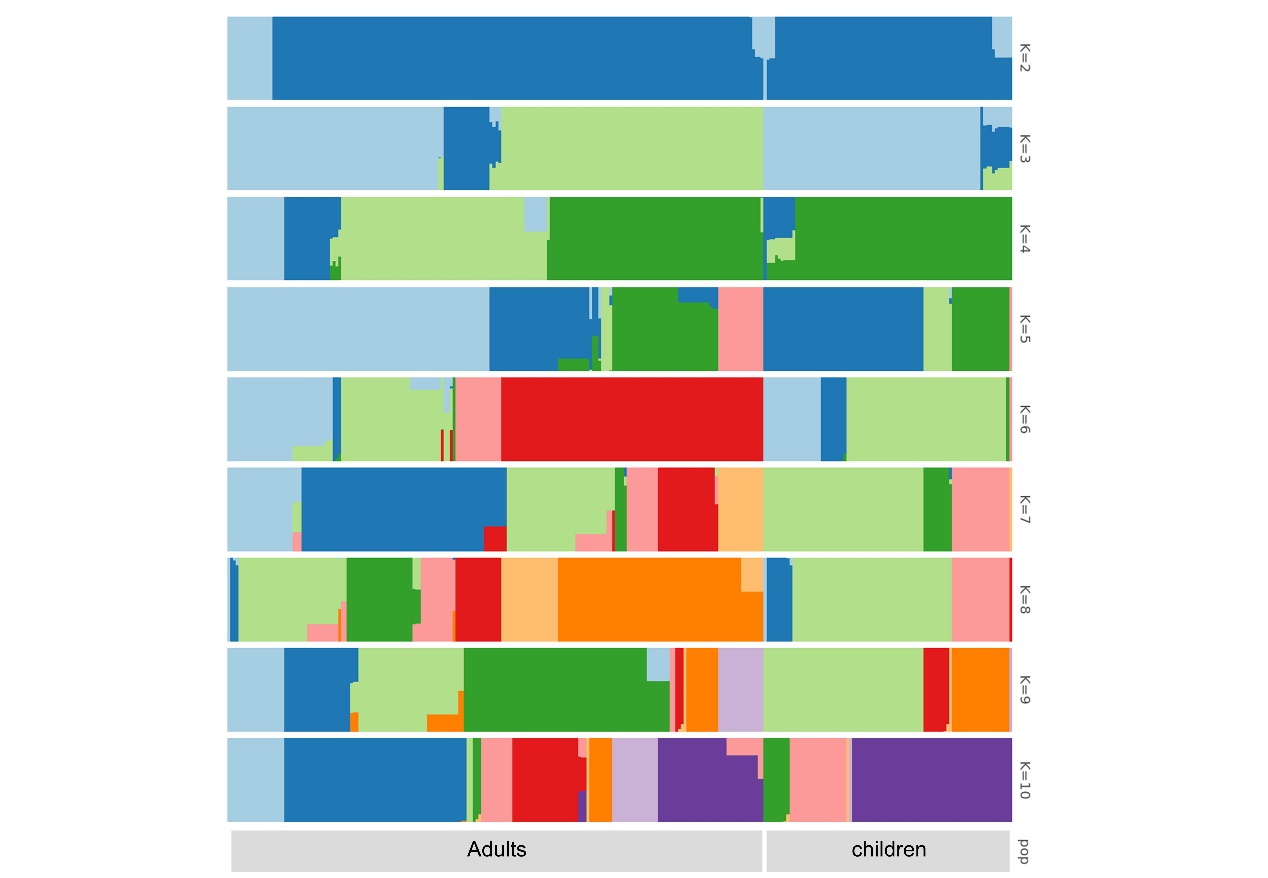


**Fig. S4** Population structure analysis of CRKP isolates. The horizontal-colored segments within columns indicate proportional ancestry contributions from different *K* values putative ancestral populations.


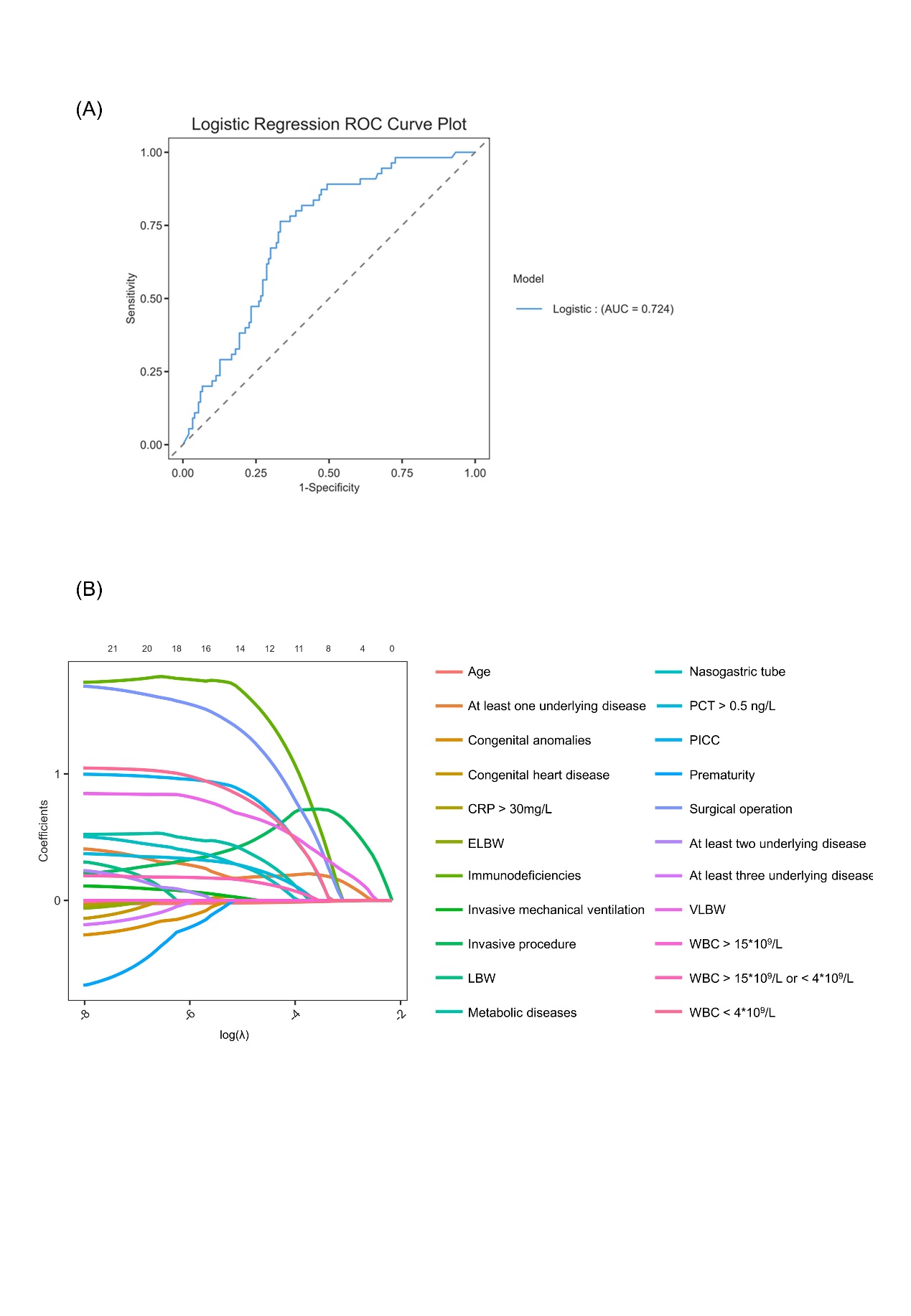


**Fig. S5** Regression analysis of risk factors associated with *iuc*^+^*rmpA2*^+^ CRKP isolation. **A** The ROC plot of multivariate binomial regression. **B** The coefficients values of Lasso regression. The shrinkage to zero demonstrates feature selection. Optimal λ (λ = 4) yields parsimonious model with four predictors.
